# Ghrelin Acylation—A Post-Translational Tuning Mechanism Regulating Adult Hippocampal Neurogenesis

**DOI:** 10.3390/cells11050765

**Published:** 2022-02-22

**Authors:** Martina Sassi, Alwena H. Morgan, Jeffrey S. Davies

**Affiliations:** Molecular Neurobiology, Institute of Life Sciences, School of Medicine, Swansea University, Swansea SA2 8QA, UK; martina.sassi@swansea.ac.uk (M.S.); a.h.morgan@swansea.ac.uk (A.H.M.)

**Keywords:** acyl-ghrelin, unacyl-ghrelin, APT1, BChE, neurodegeneration, dementia, neurogenesis

## Abstract

Adult hippocampal neurogenesis—the generation of new functional neurones in the adult brain—is impaired in aging and many neurodegenerative disorders. We recently showed that the acylated version of the gut hormone ghrelin (acyl-ghrelin) stimulates adult hippocampal neurogenesis while the unacylated form of ghrelin inhibits it, thus demonstrating a previously unknown function of unacyl-ghrelin in modulating hippocampal plasticity. Analysis of plasma samples from Parkinson’s disease patients with dementia demonstrated a reduced acyl-ghrelin:unacyl-ghrelin ratio compared to both healthy controls and cognitively intact Parkinson’s disease patients. These data, from mouse and human studies, suggest that restoring acyl-ghrelin signalling may promote the activation of pathways to support memory function. In this short review, we discuss the evidence for ghrelin’s role in regulating adult hippocampal neurogenesis and the enzymes involved in ghrelin acylation and de-acylation as targets to treat mood-related disorders and dementia.

## 1. Introduction

The gut hormone ghrelin was identified as the natural ligand of the growth hormone secretagogue receptor 1a (GHS-R1a) in 1999 [1]. It is often referred to as the “hunger hormone” thanks to its ability to stimulate food intake and regulate energy homeostasis [2]. In the years since its discovery, ghrelin has been implicated in a variety of other physiological responses linked with homeostasis, including the regulation of gastric acid secretion and motility [3], pancreatic cell proliferation and apoptosis [4], sleep [5], and cardiovascular function [6]. Notably, in the central nervous system (CNS), ghrelin has been reported to regulate action potential firing [7], mitochondrial function [7,8], immune signalling [9], neuron survival [7,10], and neural stem/progenitor cell (NSPC) differentiation into adult-born hippocampal granule cells [11,12,13].

Human ghrelin, encoded by the GHRL gene, generates the 117-amino-acid preproghrelin peptide that is enzymatically processed to the native 28-amino-acid peptide, unacyl-ghrelin (UAG). UAG undergoes enzymatic modification in the endoplasmic reticulum by ghrelin-O-acyl transferase (GOAT) to generate acyl-ghrelin, the so-called active form of the hormone [14,15]. This post-translational modification includes the addition of a medium-chain fatty acid (generally octanoic acid) at Serine residue 3 (Ser3), which is essential to bind and activate GHS-R1a signalling [16]. Notably, ghrelin is the only known peptide that requires a fatty acid modification to regulate binding to its cognate receptor. In the Golgi apparatus, either the acylated or unacylated forms of proghrelin are cleaved by the prohormone convertase 1 and 3 (PC1/3) [17]. The N-terminal of ghrelin peptide is cleaved to produce the mature 28-amino-acid form of ghrelin [17], while the C-terminal peptide is generated to form C-ghrelin, which results in the production of the hormone, obestatin [18,19].

## 2. Central Actions of Ghrelin

As ghrelin is not produced in the brain [1,20,21,22], its effects in the CNS are due to the actions of peripherally derived ghrelin. Indeed, this hypothesis is consistent with ghrelin’s ability to cross the blood–brain barrier (BBB) [7,20,23,24] and bind GHSR-1a in the brain [7,20]. From the circulation, ghrelin can cross the BBB either as acyl-ghrelin or UAG, which can be re-acylated on-site by ghrelin-O-acyl transferase (GOAT) activity, permitting fine modulation of its activity in a cell-specific manner [25,26].

The generation of new functional neurons from adult NSPCs [27,28,29,30,31] is essential for hippocampal-dependent pattern separation memory, which is the ability to discriminate between very similar contexts [32,33]. A decline in the ability to perform pattern separation is associated with age-related cognitive decline and anxiety-related behaviour, and is therefore of considerable interest to the fields of neuroscience and neurology. Notably, environmental factors such as exercise and enriched home cages for rodents enhance NSPC proliferation [34] and the differentiation and survival [35] of new adult-born neurones, respectively. These phenomena have led to the identification of several factors generated outside of the CNS that regulate adult hippocampal neurogenesis (AHN), including irisin [36], klotho [37], cathepsin B [38], clusterin [39], eotaxin [40], growth differentiation factor11 (GDF11) [41], β_2_ microglobulin (B2M) [42], and glycosylphosphatidylinositol-specific phospholipase D1 (Gpld1) [43]. Similarly, calorie restriction increases AHN primarily by supporting differentiation and survival [44] via ghrelin signalling [12,45]. The precise molecular mechanisms underpinning these pro-neurogenic effects require further elucidation; however, soluble neurotrophic factors, such as brain-derived neurotrophic factor (BDNF), are known to promote AHN [46]. In a recent study from our lab, we examined whether acyl-ghrelin stimulated the production and/or the release of such factors. Firstly, BDNF gene expression was quantified after treating primary hippocampal cells with acyl-ghrelin. BDNF mRNA was significantly increased after acyl-ghrelin treatment and the survival of new-born cells was increased in a BDNF-dependent mechanism [47]. These findings are consistent with previous in vitro studies demonstrating increased BDNF in ghrelin-treated spinal cord neurones [48] and evidence that ghrelin increases BDNF levels in the mouse hippocampus [49]. To assess the acyl-ghrelin effect in vivo, adult wild-type mice were intraperitoneally injected with acyl-ghrelin and, 24 h later, the brains were analysed using in situ hybridisation to show that acyl-ghrelin significantly increased BDNF IXa mRNA, in the rostral granule cell layer (GCL) of the dentate gyrus (DG). This, to our understanding, is the first demonstration that acyl-ghrelin regulates BDNF specifically within the neurogenic niche of the hippocampus’ GCL. A more recent study supports the notion that ghrelin regulates BDNF mRNA from hippocampal homogenates collected from ex-vivo-treated tissue [50]. In this study, the authors note that ghrelin increased BDNF mRNA in a promoter- and age-specific manner, suggesting that further in situ hybridisation or spatial transcriptomic approaches are warranted to delineate the cell-specific molecular mechanisms that support ghrelin-mediated neurogenesis. To show that acyl-ghrelin regulates secreted BDNF protein, we treated NSPCs, which do not express GHS-R1a, with conditioned media from acyl-ghrelin-treated primary hippocampal cultures and reported a significant increase in the number of surviving new-born cells. This effect was completely blocked by the addition of a BDNF-neutralising antibody, suggesting that acyl-ghrelin supports the survival of new-born hippocampal cells via increased BDNF signalling [47].

Collectively, the above studies are in line with acyl-ghrelin stimulating the hippocampal BDNF pathway and are consistent with previous reports linking BDNF, AHN, and pattern separation memory [46]. However, little was known about the neurogenic role of UAG, the most prevalent form of circulating ghrelin. As mentioned earlier, UAG was considered biologically inactive as it lacks the acyl-motif necessary for binding GHS-R1a. Despite this, reports suggested that it stimulated genome-wide changes in gene expression independently of the ghrelin receptor [51] and blocked acyl-ghrelin-induced food intake [52]. To shed light on whether UAG regulated AHN, hippocampal cell cultures co-treated with acyl-ghrelin and UAG at equimolar (1:1) and non-equimolar doses (1:10 and 1:30) demonstrated that UAG inhibited the pro-survival effect of acyl-ghrelin at each of the ratios tested. Consistent with this finding, UAG-treated WT and GOAT^−/−^ mice had significantly fewer proliferating cells and immature neurons in the DG of the hippocampus. Moreover, in GOAT^−/−^ mice, which lack acyl-ghrelin but have high levels of UAG, the number of immature neurons was also significantly reduced after treatment with saline, suggesting that UAG can regulate AHN independently of acyl-ghrelin via an unknown mechanism. In addition, the reduction in new non-neuronal adult-born cells in both UAG-treated WT mice and GOAT^−/−^ mice suggested that UAG may regulate new astroglia or new NSPCs in the hippocampus.

The alterations in markers of new adult-born neurons were accompanied by diminished plasticity-related markers—c-Fos expression and dendritic spines—and with impaired performance on the hippocampal-dependent spatial memory Y-maze task in GOAT^−/−^ mice. However, treatment of GOAT^−/−^ mice with acyl-ghrelin once daily for 7 days restored performance to WT levels [47]. These data suggest that acyl-ghrelin signalling is activating both neurogenic and other hippocampal pathways to support memory function and is consistent with previous studies identifying enhanced hippocampal long-term potentiation (LTP) [7,53,54], increased expression of GluA1-containing amino-3-hydroxy-5-methyl-4-isoxazolepropionic acid (AMPA) receptors [55], as well as the phospho-GluN2B [56] and the NR2B subunit of the N-methyl-D-aspartate receptor (NMDA) receptor [57], in response to acyl-ghrelin. Our findings support a role for UAG in blocking acyl-ghrelin-mediated GHS-R1a signalling in the hippocampus [47]. Could this also involve UAG modulating other plasticity mechanisms, such as LTP, AMPA, and NMDA receptor function, in the hippocampus? These studies are warranted to further our understanding of ghrelin-regulated hippocampal plasticity.

Collectively, these findings suggest that reducing UAG and increasing acyl-ghrelin in the blood may lead to the promotion of hippocampal plasticity and pro-neurogenic signalling. Modulation of the acyl-ghrelin to UAG (AG:UAG) ratio may be relevant to disorders associated with impairments in AHN and hippocampal-dependent behaviours. Indeed, acyl-ghrelin ameliorates hippocampal neuroinflammation, nerve cell loss, and cognitive decline in rodent models of neurodegenerative disorders, including Alzheimer’s disease (AD) [58]. In the 5xFAD mouse (express human Amyloid precursor protein and Presenilin-1 transgenes with a total of five AD-linked mutations), acyl-ghrelin increased the number of immature hippocampal neurons, albeit without reducing amyloid beta deposits [59]. The anxiolytic effect and pro-neurogenic effect of calorie restriction was also dependent on acyl-ghrelin signalling [60], suggesting that mood-related disorders linked with impairments in new adult-born neuron function may benefit from an increase in the AG:UAG ratio.

## 3. Neurogenesis in Humans—Relevance to Ghrelin Signalling

The quantification of AHN in post-mortem human tissues is often restricted due to the sub-optimal methods used for brain tissue collection and preservation. However, recent studies using an optimised protocol for the collection, fixation, and immunofluorescent detection of native proteins expressed in NSPCs, neuroblasts, and new neurons [61] reveal that AHN is impaired in AD [30] and other age-related neurodegenerative diseases [31]. In this study, diseases associated with impaired AHN included the alpha-synucleinopathies, dementia with Lewy bodies, and Parkinson’s disease (PD). PD dementia (PDD) has been linked with impairments in NSPC division and hippocampal immature neuron formation [62], as well as poor pattern separation memory performance [63]. Our analysis of plasma samples collected from individuals diagnosed with PDD demonstrated a reduction in the AG:UAG ratio, compared to cognitively intact PD and control groups. On first inspection, these plasma data deviate from previous reports describing a reduction in circulating acyl-ghrelin in PD subjects [64,65]. However, this difference may be due to the stratification of PD groups by cognitive performance and our reporting of the AG:UAG ratio rather than acyl-ghrelin alone. We also reported that the levels of octanoic acid (C8:0), the predominate substrate for the acylation of ghrelin, were similar among the three experimental groups [47]. Therefore, the reduction in the AG:UAG ratio seems to be caused by the altered activity of ghrelin acylation and/or de-acylation enzymes, rather than by reduced octanoic acid. These findings in humans diagnosed with PDD support the position that elevating AG:UAG levels may restore pro-neurogenic signalling and hippocampal function. However, for such an approach to be effective, a wider pathway supporting this complex circuitry would also need to remain intact. To this end, we reported that the expression of GHSR-1a mRNA in the adult human hippocampal GCL remained unaltered across the three experimental groups tested [47]. As GHSR-1a is expressed in the hippocampus of PDD patients, an increase in the AG:UAG ratio may promote the activation of pro-neurogenic GHSR-1a signalling. As mentioned, GHSR-1a activation has been linked with promoting neurogenesis as well as hippocampal synaptic and memory function [7,13,55,60,66]. However, impairments in the GHS-R1a pathway were recently described in the 5xFAD mouse model of familial AD that were overcome by co-treatment with GHS-R1a and Drd1 agonists [67]. Interestingly, GOAT protein expression was significantly decreased in crude human hippocampal lysate from both PD and PDD brains, when quantified using Western blot. Moreover, the number of cells immunopositive for GOAT within the DG of the hippocampus was significantly reduced in the PDD brain relative to the control and PD groups [47].

Together, the reduction in both plasma AG:UAG ratio and hippocampal GOAT may lead to attenuated GHS-R1a signalling in the hippocampus and contribute to the cognitive deficits in PDD.

## 4. Targeting the AG:UAG Ratio

Several distinct ghrelin-receptor agonists have been developed for the treatment of disorders associated with reductions in lean mass or gastric motility. For example, the GHS-R1a agonist, anamorelin, was given regulatory approval in Japan as a new therapy for cachexia [68]. Notably, the efficacy of these compounds in models of neurodegeneration has raised the possibility of drug re-purposing to treat CNS-related disorders [58,69,70,71,72]. The GHS-R1a agonist, MK-0677, when administered alongside the Drd1 agonist, SKF81297, restored synaptic function in the 5xFAD mouse without affecting amyloid plaques [67]. Meanwhile, a higher dose of MK-0677 ameliorated Aβ deposition, neuroinflammation, and neurodegeneration in the 5xFAD mouse model [71] and promoted the accumulation of a AMPA receptor on excitatory hippocampal synapses and improved LTP [55]. However, a clinical trial of MK0677 reported no clinical effect on disease progression in AD patients [73]. The GHS-R1a agonist, LY444711, improved spatial memory performance in the APPSwDI mouse model of AD without altering glucose metabolism [72], suggesting that this treatment may be well tolerated in AD patients, who often present with impaired glucose metabolism [74]. Therefore, GHS-R1a agonists may be an appropriate target to treat neurodegenerative disorders and diseases affecting cognition. Further work is necessary to determine their effects on AHN and pattern-separation-dependent memory, particularly in models of age-related neurodegeneration [75].

Despite their promise, synthetic GHS-R1a agonists are unlikely to address the inhibitory action of UAG on AHN and hippocampal-dependent memory. The level of circulating acyl-ghrelin depends on its rate of synthesis as well as enzymatic processing. In 2004, the acyl-ghrelin half-life in serum was reported to vary between humans (240 min) and rats (30 min), suggesting that, unlike ghrelin acylation—which is performed uniquely by GOAT—several enzymes may be involved in its de-acylation [76].

The first enzyme reported to promote the de-acylation of acyl-ghrelin in human serum was the serine esterase Butyrylcholinesterase (BChE). However, treatment with the BChE inhibitors eserine salicylate and sodium fluoride only partially inhibited the reaction, suggesting that it was not the only esterase responsible for ghrelin de-acylation [76]. More recently, a correlation between BChE over-expression in rodents and decreased acyl-ghrelin plasma levels was reported [77]. BChE is expressed in the human hippocampus, with increased expression reported in the AD brain [78]. Thus, increased activity of BChE—if confirmed—could decrease the AG:UAG ratio, which is linked with PDD [47]. In 2004, de-acylation of acyl-ghrelin in rat stomach homogenate was reportedly mediated by acyl-protein thioesterase 1 (APT1) [79]. Subsequently, both APT1 and its close homologue, APT2, were reported to catalyse the de-acylation of ghrelin in murine macrophage (RAW264.7) and liver hepatocellular carcinoma (HepG2) cells. HepG2 cells released functional APT1—but not APT2—into the media, suggesting that APT1 is secreted and may de-acylate substrates in an endocrine manner, while APT2 activity may be restricted to the intracellular space [80]. Notably, an increase in APT1 protein expression in the hippocampus of aged mice correlated with decreased performance in the novel object recognition test, suggesting that APT1 may play a role in age-related memory function [81]. In addition to de-acylating acyl-ghrelin, APT1 de-palmitoylates PSD-95 [82] and huntingtin [83]. These studies report that pharmacological inhibition of APT1 ameliorates disease phenotypes associated with AD and Huntington’s disease (HD), respectively. Furthermore, another recent study [84] demonstrated that inhibition of APT1 reversed neuropathology, locomotor deficits, and anxio-depressive behaviours in HD knock-in mice. Therefore, whilst the precise mechanism of action of APT1 inhibitors remains unclear, they represent a novel therapeutic approach to treat neurodegenerative diseases and mood-related disorders.

In addition to BChE and APT1, enzymes such as APT2 [80] and α2-macroglobulin [85] have been shown to de-acylate acyl-ghrelin, while others, such as platelet-activating factor (PAF), have also been proposed [79]. Moreover, a carboxylesterase inhibitor (bis-p-nitrophenyl-phosphate) completely inhibited ghrelin de-acylation, indicating a link between ghrelin de-acylation and carboxylesterase activity [76]. Indeed, Notum, a carboxylesterase known for de-acylating proteins involved in the Wnt signalling pathway [86], de-acylates ghrelin [87]. Targeting the activities of these enzymes represents a potentially novel approach to regulate the AG:UAG ratio with, perhaps, relevance to human disease.

## 5. Conclusions

As diagnostic biomarkers play an important role in characterising disease onset and progression, the need for blood-based markers to complement the expensive and invasive cerebrospinal fluid (CSF)-based and positron emission tomography (PET)-based biomarkers such as β-amyloid, tau, and neurofilament light chain are eagerly anticipated in dementia [88]. The newly discovered role of UAG in reducing AHN, combined with the fact that its acylated counterpart has been shown to exert an opposing—beneficial—effect, may be important in the search for novel blood-based biomarkers of dementia. In the future, longitudinal studies that clarify whether AG:UAG represents a prognostic dementia biomarker are warranted. Moreover, larger cohorts of clinically distinct groups are required to further delineate the role of ghrelin in other forms of neurodegeneration and dementia.

In neurodegenerative disorders, the loss of existing developmentally born neurones is often combined with impaired AHN, indicating that the capacity for cell renewal is compromised or lost [89]. Recently, several studies focused on the ability of acyl-ghrelin to promote AHN and memory in rodent models of health and disease [75,90,91,92] (see Table 1). Indeed, promoting GHS-R1a signalling, using synthetic agonist compounds, or inhibiting ghrelin de-acylation enzymes may be a valuable tool in promoting hippocampal ghrelin–GHS-R1a signalling, offering novel therapies to ameliorate cognitive decline and mood-related disorders in humans. Moreover, a novel role for UAG in the context of AHN is evident from the pre-clinical and clinical data reported [47], supporting the hypothesis that UAG blocks acyl-ghrelin-mediated GHS-R1a signalling in the hippocampus resulting in impaired hippocampal plasticity and neurogenesis.

The increasingly recognised role of ghrelin as an important modulator of AHN and memory function [75] comes at a time when aberrant neurogenesis is firmly implicated in age-related cognitive impairment and neurodegenerative disorders in humans [30,31]. We suggest that the relationship between ghrelin acylation and AHN may be relevant to brain ageing, mood-related disorders, and dementias—warranting further investigation in humans.

## Figures and Tables

**Table 1:** Studies analysing the effect of ghrelin peptide adult hippocampal neurogenesis. Abbreviations: GH = growth hormone, KO = knock-out, WT = wild type, 6-OHDA = 6-hydroxydopamine, CUMS = chronic unpredictable mild stress.

Model & Methods	Outcome	Reference
Ghrelin KO mice (8–9 weeks of age) given acyl-ghrelin for 8 days.	Increased proliferation and neuronal differentiation.	[66]
GH-deficient spontaneous dwarf rats (8–12 weeks of age) given acyl-ghrelin for 8 days.	Increased proliferation and neuroblast number.	[90]
5xFAD mice (8 weeks of age) given acyl-ghrelin every 2 days for 30 days.	Increased proliferation and neuroblast number.	[20]
C57Bl/6NCrlVr mice (8–10-weeks of age) given acyl-ghrelin for 8 days.	Increased new immature neurone and mature neurone number.	[91]
Lister hooded rats i.p. injected with acyl-ghrelin for 14 days.	Increased neuroblast and mature neurone number. No change in stem cell self-renewal.	[13]
Sprague Dawley rats with 6-OHDA lesion i.p. injected with acyl-ghrelin for 8 weeks.	Increased neuroblast number.	[11]
7–8 weeks old male CUMS mice (on C57BL/6 background) i.p injected with acyl-ghrelin once a day for 2 weeks.	Increased proliferation and new immature neurone number.	[92]
UAG-treated WT and GOAT^−/−^ mice	Decreased proliferation, neuroblasts, new immature neurones. Decreased new non-neuronal cells.	[47]
